# A technique for pediatric chest wall reconstruction using custom-designed titanium implants: description of technique and report of two cases

**DOI:** 10.1007/s11832-015-0709-1

**Published:** 2016-01-18

**Authors:** Colin J. Anderson, Murray D. Spruiell, Erin F. Wylie, Caitlin M. McGowan, Frederic W. -B. Deleyiannis, Nathan J. Donaldson, Travis C. Heare

**Affiliations:** Department of Orthopaedic Surgery, University of Colorado, Anschutz Medical Campus, 12631 E. 17th Avenue, Mail Stop B202, Aurora, CO 80045 USA; Department of Orthopaedic Surgery, Musculoskeletal Research Center, Children’s Hospital Colorado, Anschutz Medical Campus, 13123 East 16th Avenue, B060, Aurora, CO 80045 USA; Department of Plastic and Reconstructive Surgery, Children’s Hospital Colorado, Anschutz Medical Campus, 13123 East 16th Avenue, B467, Aurora, CO 80045 USA

**Keywords:** Chest wall reconstruction, Chest wall resection, Chest wall deficiency, Poland’s syndrome, Pediatric orthopedics

## Abstract

**Purpose:**

We present a surgical technique for chest wall reconstruction using custom-designed titanium implants developed for two female patients to provide both chest wall symmetry and adequate stability for staged breast reconstruction.

**Methods:**

A retrospective review was performed for two adolescent female patients with large chest wall defects who underwent the described technique. The etiology of the chest wall deficiency was secondary to Poland’s syndrome in one patient, and secondary to surgical resection of osteosarcoma in the other patient. For each patient, a fine-cut computed tomography scan was obtained to assist with implant design. After fabrication of the prosthesis, reconstruction was performed though a curvilinear thoracotomy approach with attachment of the implant to the adjacent ribs and sternum. Wound closure was obtained with use of synthetic graft material, local soft tissue procedures, and flap procedures as necessary.

**Results:**

The two patients were followed post-operatively for 35 and 38 months, respectively. No intra-operative or post-operative complications were identified. Mild scoliosis that had developed in the patient following chest wall resection for osteosarcoma did not demonstrate any further progression following reconstruction.

**Conclusions:**

We conclude that this technique was successful at providing a stable chest wall reconstruction with satisfactory cosmetic results in our patients.

## Introduction

The chest wall serves the vital structural and functional purposes of protecting the intra-thoracic organs, promoting respiration, and supporting the actions of the upper extremities [1]. Given its significant physiologic importance, chest wall deficiency has the potential to cause devastating morbidity and even life-threatening consequences. In cases of chest wall deficiency, surgical reconstruction is indicated to improve structural integrity, respiratory function, and unsatisfactory cosmetic appearance [2–4].

A variety of etiologies may result in chest wall deficiency ranging from acquired conditions to congenital deformities [1, 4]. While only 1.8 % of solid tumors in children occur in the chest wall, a majority of the tumors are malignant [5]. Due to the necessity for wide-surgical excision, chest wall resection for sarcoma often results in defects of significant size. Acquired defects may also result from debridement of necrotic or fibrotic tissue secondary to the sequelae of radiation therapy, traumatic injuries, or infection. In addition to acquired conditions, there are a variety of congenital disorders that also cause chest wall deformity. Of particular interest with respect to chest wall reconstruction, Poland’s syndrome is a rare disorder characterized by the absence of multiple ribs, the absence of pectoralis musculature, and hypoplasia or aplasia of the breast or nipple, that is capable of producing significant chest wall deficiencies [4, 6].

Despite the myriad operative procedures currently utilized for chest wall reconstruction, no consensus exists on an optimal surgical strategy. The purpose of this study is to present a reconstruction technique using custom-designed titanium implants that were developed with the goals of improving chest wall stability as well as cosmetic results over currently described techniques. We present two cases of adolescent females with prominent cosmetic concerns due to chest wall and breast asymmetry for which this technique was developed.

## Surgical technique

A fine-cut thoracic computed tomography scan is obtained and three-dimensional pre-operative planning is performed for prosthetic design. The intact contralateral ribs are mirrored to the side of the defect to provide a template for shaping thoracic symmetry. A custom prosthesis is designed to reconstruct the defect with attachments to adjacent ribs as well as the sternum with care taken to ensure the implant is capable of completely spanning the defect anteriorly to posteriorly. Subsequently, the custom implant is fabricated from titanium (Biomet Inc., Warsaw, IN, USA). Pre-operatively, when inadequate soft-tissue coverage is expected following placement of the implant, consultation with plastic surgery should be obtained to plan for skin or flap coverage needs.

At the time of surgery, the patient is placed in the lateral decubitus position, and the surgical field is draped to include the sternum anteriorly and the spine posteriorly. A curvilinear thoracotomy incision is made overlying the level of the defect. Full-thickness fasciocutaneous flaps are elevated from the chest wall, and exposure is extended anteriorly to the sternum and posteriorly to the deficient ribs. Sections of pectoralis, latissimus dorsi, or other peri-scapular musculature may require division for adequate exposure. If a sternal osteotomy is planned, a periosteal elevator is used to circumferentially expose the sternum, and osteotomy is performed with an oscillating saw protected with a malleable retractor.

Attention is then turned to fitting of the prosthesis. The anterior costal cartilages and sternum are trimmed to match the sternal component. Likewise, the deficient ribs are exposed circumferentially and trimmed to fit rib docking sites on the implant. The pleural defect may be covered with graft or mesh depending on surgeon preference. Prior to final fixation, a chest tube should be placed. Subsequently, the prosthesis is placed and secured. For fixation to the sternum, 4–5 transosseous #5 Fiberwire^®^ (Arthrex, Inc., Naples, FL, USA) sutures are placed through pre-fabricated holes in the implant. The first implant design (described in Case 1 below) utilized tubes with locking trap-doors that provided secure fixation to the ribs. However, due to prominence on the chest wall post-operatively, the second prosthesis (described in Case 2) was designed with smaller rib docking sites where two transosseous #5 Fiberwire^®^ sutures are placed through pre-fabricated holes for fixation to the ribs. For closure, the sectioned posterior musculature is approximated with reduction of the scapula. The subcutaneous tissue is closed with interrupted sutures and the skin is closed with a running subcuticular suture. The wound is sterilely dressed and the chest tube placed to vacuum suction. Post-operative analgesia is provided with an epidural pain catheter.

## Case 1

**Fig. 1:**
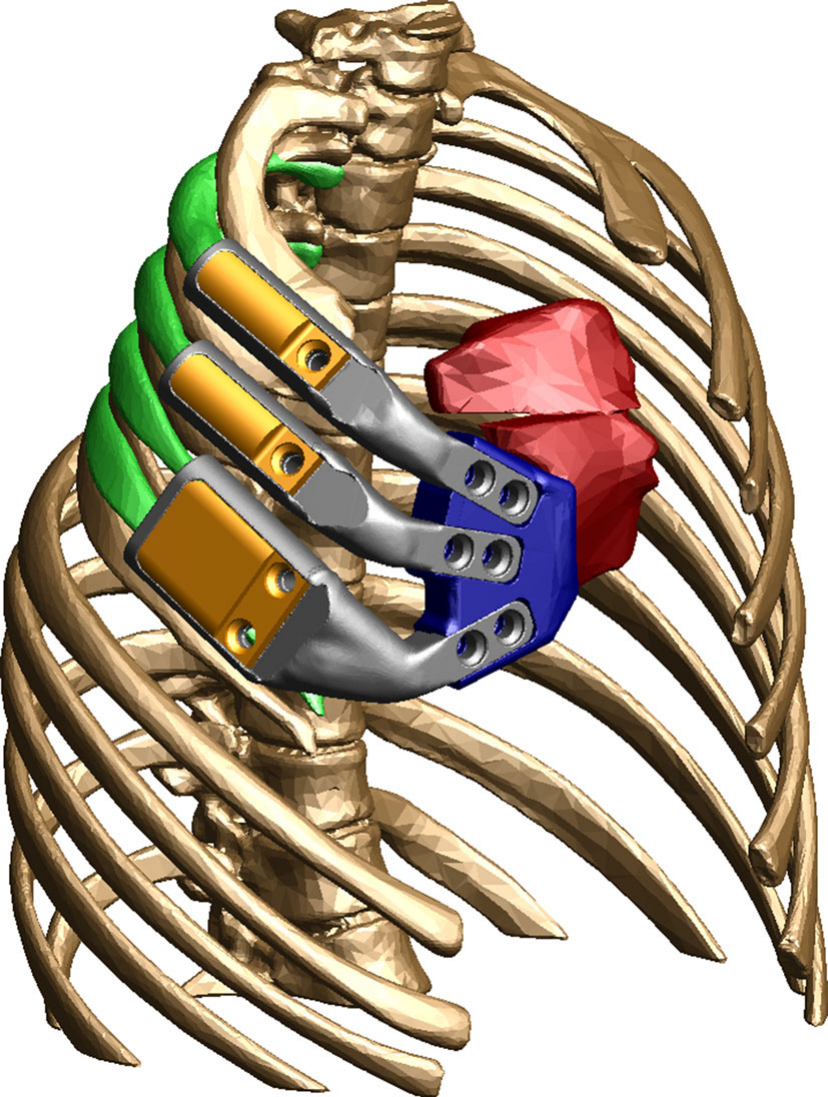
Oblique view of the three-dimensional model of the prosthesis for reconstruction of the chest wall defect secondary to Poland’s syndrome. *Colors* represent osteotomized sternum (*red*), sternal plate (*blue*), prosthetic ribs (*gray and orange*), and repositioned second through fifth ribs (*green*)

**Fig. 2:**
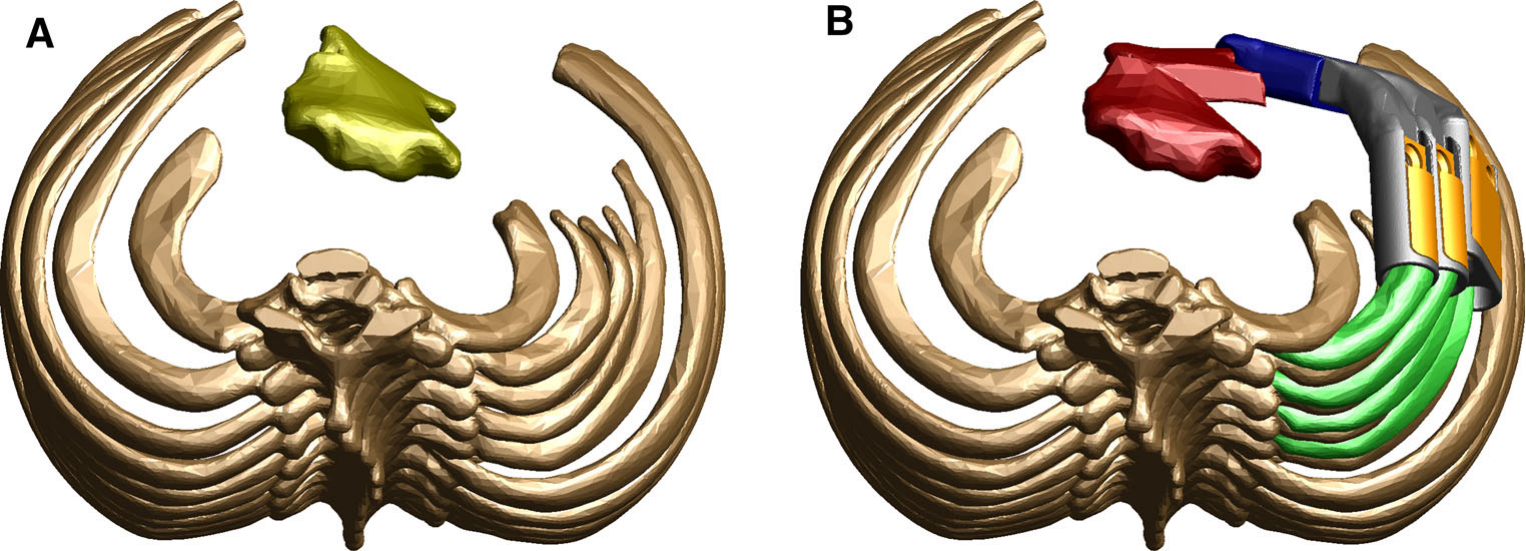
Superior view of three-dimensional model demonstrating the difference between **a** the native sternum (*yellow*) and rib position, and **b** the reconstructed symmetrical chest wall after sternal osteotomy and implant placement

**Fig. 3:**
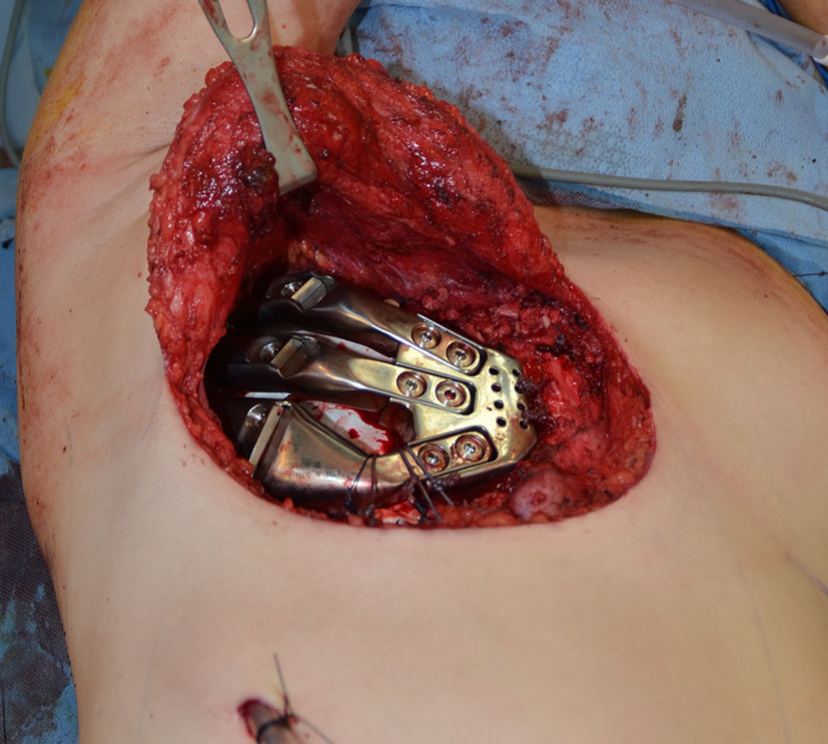
Intra-operative photograph showing placement of the prosthesis with FiberWire^®^ sutures securing the prosthesis to the sternum anteriorly and to the sixth rib inferiorly. The Gore-tex^®^ graft is visible underneath the prosthesis, and the chest tube is located inferiorly. Image oriented with anterior to the *right* and superior to the *top*

**Fig. 4:**
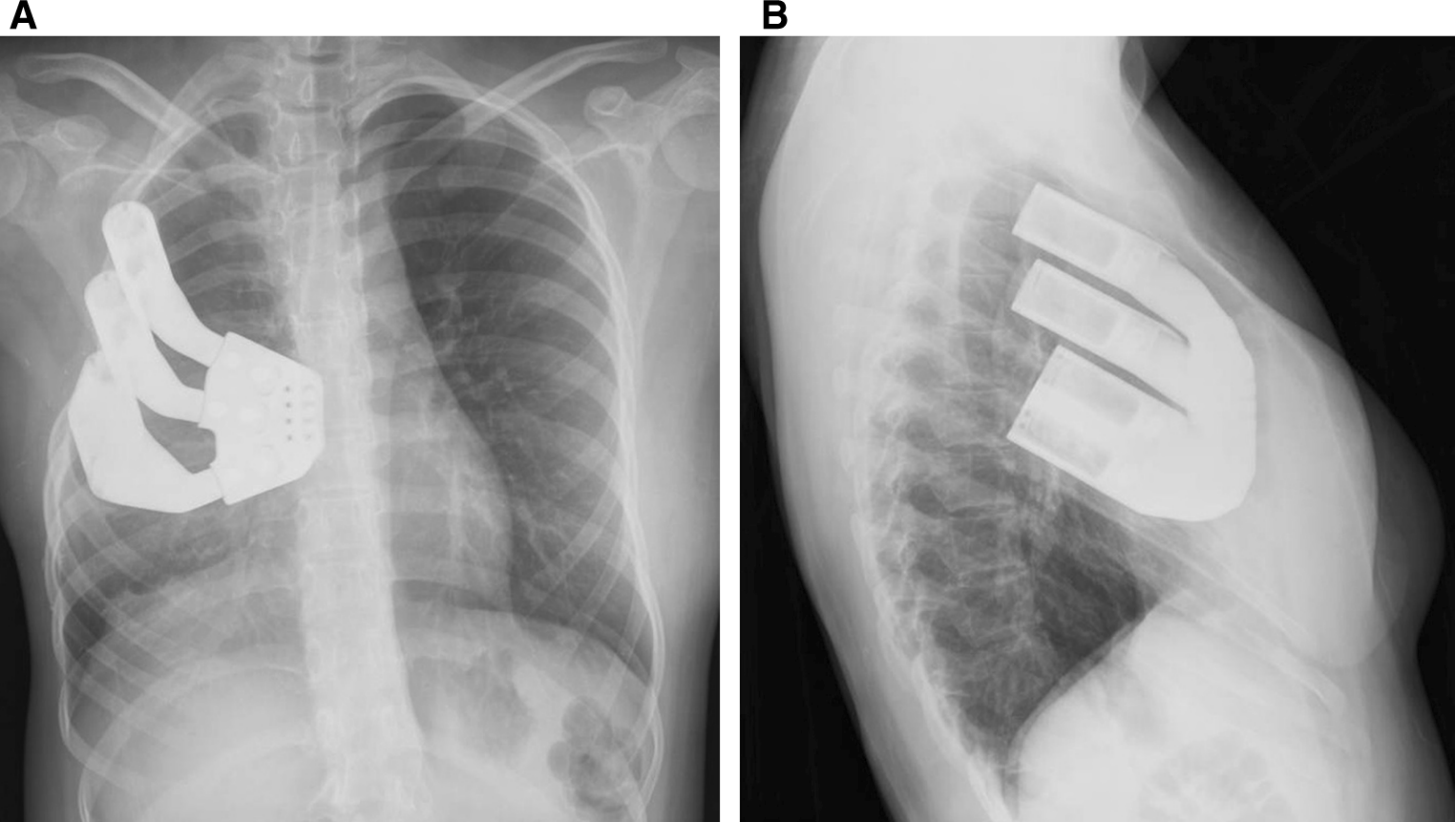
Post-operative **a** anteroposterior and **b** lateral radiographs demonstrating stable position of the prosthesis

A 15-year-old girl with a history significant for congenital chest wall deficiency secondary to Poland’s syndrome presented for evaluation due to cosmetic concerns and desire for breast reconstruction. Examination of the chest demonstrated athelia and amastia, absence of the pectoralis and serratus musculature on palpation, and anterior deficiency of the second through fifth ribs with associated paradoxical respiratory movement. Brachysyndactyly of the ipsilateral small finger was also noted. For reconstruction, the custom prosthesis was designed with planned derotational osteotomy of the sternum to allow for adequate thoracic expansion (Fig. 1). Volumetric evaluation demonstrated a theoretical increase in lung volume of 32 % (Fig. 2). However, pre- and post-operative pulmonary function test data were not obtained. The procedure was performed as described above (Figs. 3, 4). Closure was performed with a latissimus myocutaneous rotational flap performed in coordination with plastic surgery.

There were no observed intra-operative or post-operative complications. Eight months after the procedure, breast reconstruction was initiated with placement of a tissue expander between the latissimus flap and the titanium prosthesis, followed 5 months later by definitive breast implant placement. Fifteen months post-chest wall reconstruction, the patient underwent right nipple reconstruction and fat augmentation, with additional fat augmentation performed at 23 months. At 38 months post-operatively, the patient continues to be subjectively satisfied with the results functionally and cosmetically. No evidence of rib fracture or implant subsidence has yet been identified in the follow-up period. A subset of the surgical data for this patient has been previously reported from the perspective of breast reconstruction [7].

## Case 2

**Fig. 5:**
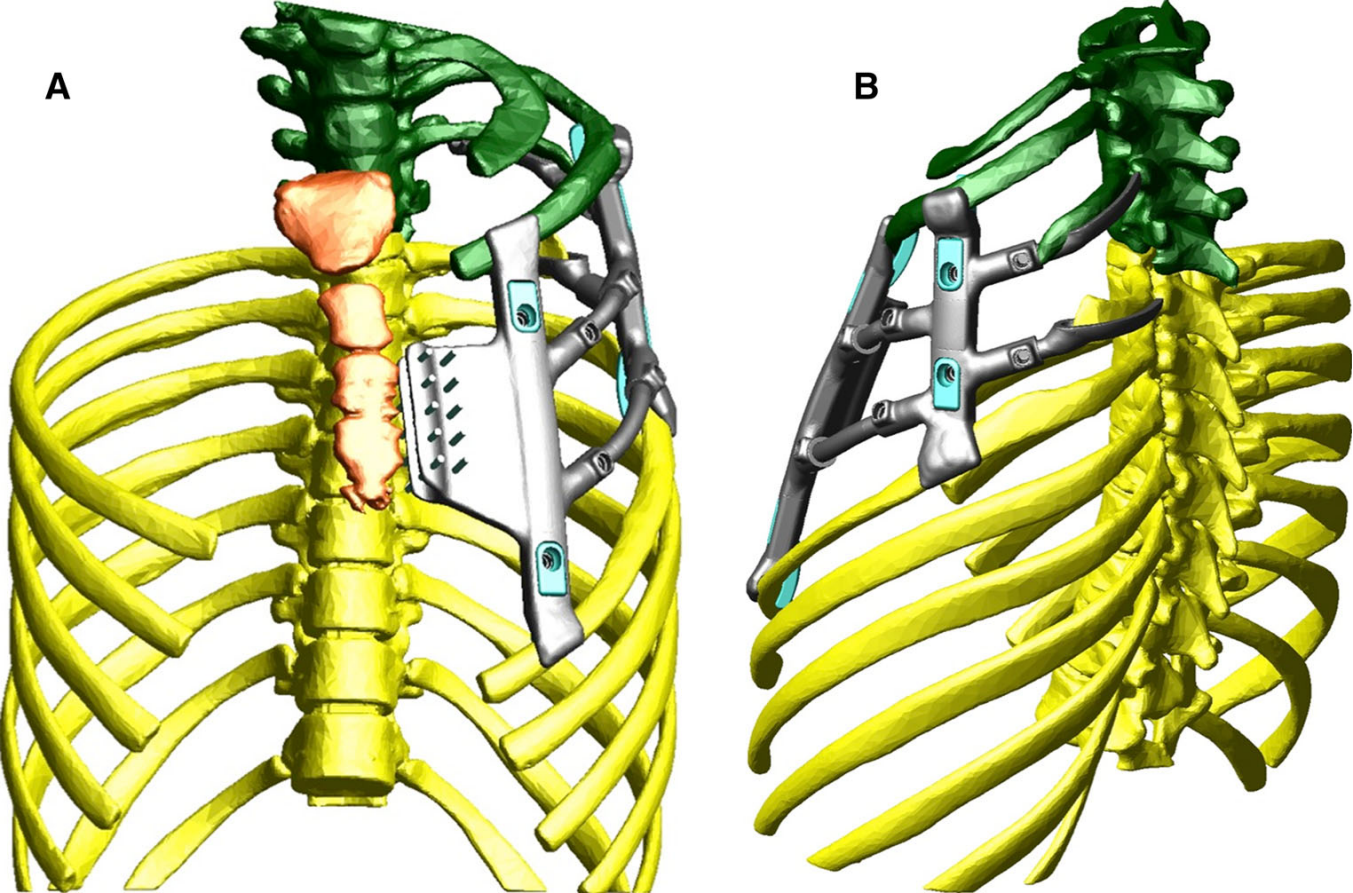
Frontal (**a**) and oblique (**b**) views of the three-dimensional model of the prosthesis for reconstruction of the chest wall defect secondary to osteosarcoma resection, designed to attach to the sternum anteriorly, to the second rib superiorly, to the sixth rib inferiorly, and to the third and fifth rib remnants posteriorly. *Colors* represent sternum (*orange*), inferior ribs and vertebra (*yellow*), superior ribs and vertebra after repositioning with the prosthesis in place (*green*), rib clips (*blue*). Suture holes shown as small rods protruding from the anterior sternal plate

**Fig. 6:**
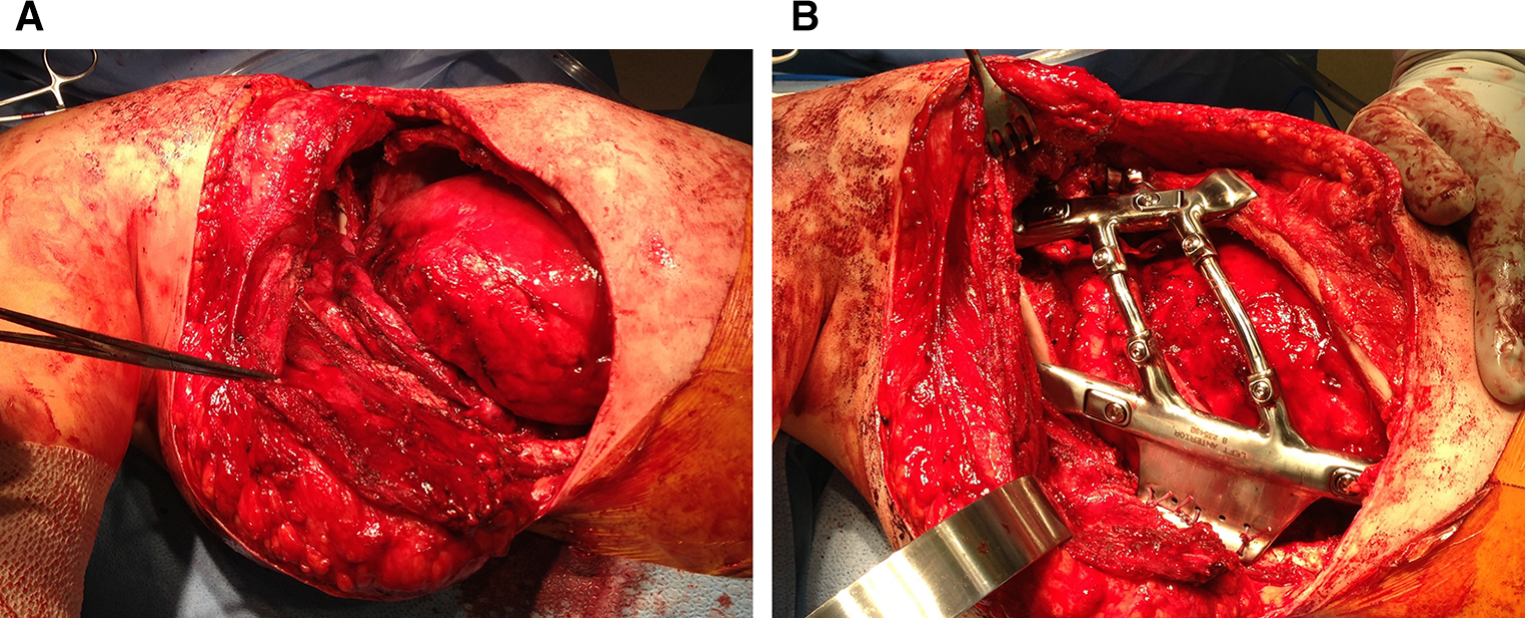
Intra-operative photographs of the patient in the* right* lateral decubitus position showing **a** chest wall defect after exposure of sternum and remnant ribs, and **b** the prosthesis secured in place. Image oriented with cranial to the *left* and anterior to the *bottom*

A 16-year-old girl with a history of left chest wall osteosarcoma status post chest wall resection 5 years earlier presented with complaints of poor cosmetic appearance due to asymmetry. The initial sarcoma resection included removal of the entire fourth as well as portions of the third and fifth ribs, with polypropylene mesh (Marlex^®^; Davol, Warwick, RI, USA) placement for closure. On examination, there was superolateral displacement of the left breast into the defect as well as hypoplasia of the nipple and areola. Lung herniation and paradoxical respiratory movement were also present. Radiographs demonstrated a levoconvex upper thoracic scoliosis measuring 20° that had developed in the 5 years since the resection. Reconstruction was planned with a custom prosthesis (Fig. 5). At the time of the reconstruction, the existing polypropylene mesh, placed during the initial resection, was found to be densely scarred to both the lung and chest wall. To obtain exposure, the mesh was elevated in continuum with the fasciocutaneous flap and breast (Fig. 6a). Next, the prosthesis was secured anteriorly to the sternum, superiorly to the second rib, inferiorly to the sixth rib, and posteriorly to the remnant third and fifth ribs (Fig. 6b). Lastly, the fasciocutaneous flap with adherent mesh was closed over the defect.

**Fig. 7:**
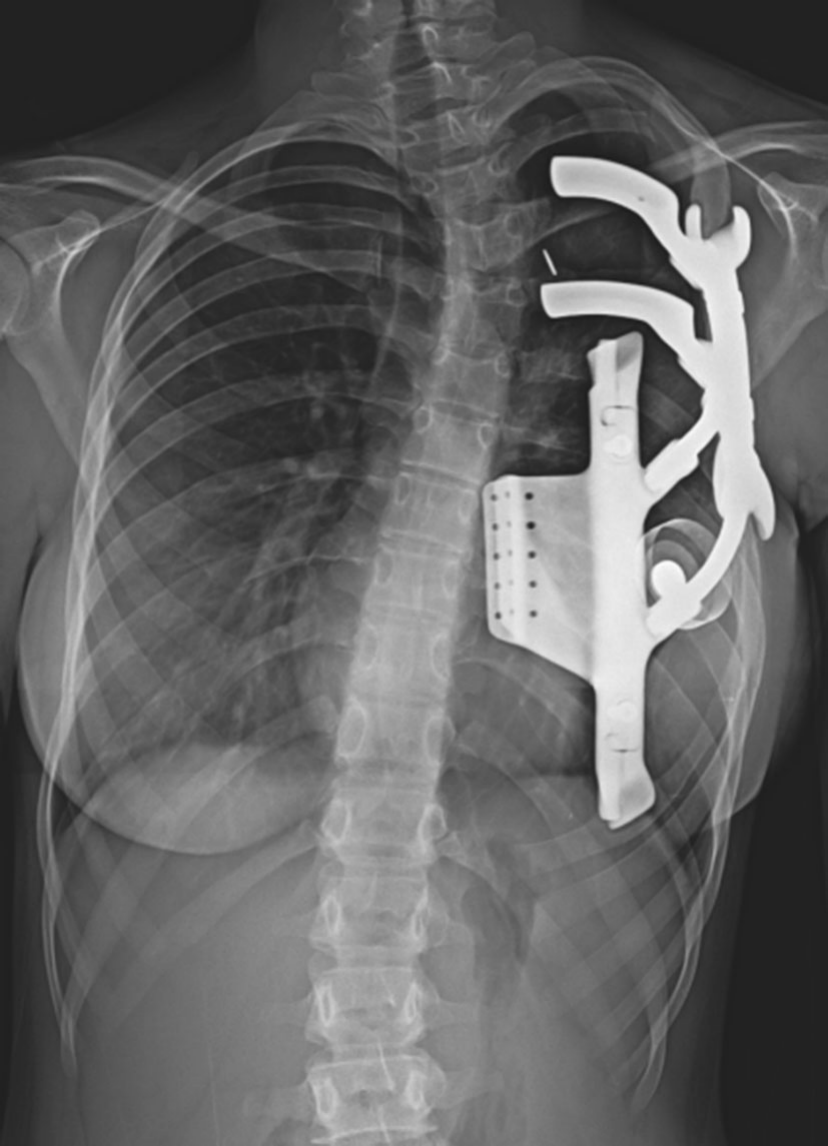
Post-operative posteroanterior radiograph demonstrating position of the prosthesis. The third rib remnant is visualized superior to the adjacent rib holster. The levoconvex upper thoracic scoliosis curve Cobb angle measures 29°

There were no observed intra-operative or post-operative complications. Immediate post-operative radiographs demonstrated post-procedural progression in the upper thoracic scoliosis to 25°, which is concluded to be a result of expansion forces on the chest wall defect by the prosthesis. No further progression has yet been noted in the follow-up period (Fig. 7). In the early post-operative period, it was noted that the posterior remnant third rib dissociated from the prosthesis with superior migration. However, continued monitoring has not demonstrated further change in position. Due to continued breast asymmetry, at 21 months post-reconstruction, the patient underwent left breast soft-tissue expander placement with definitive implant placement at 27 months. At 35 months post-operatively, the patient continues to be subjectively satisfied with the cosmetic and functional results of the chest wall reconstruction.

## Discussion

Over the past half-century, remarkable progress has been made in the treatment of chest wall deficiencies; however, the numerous operative techniques described are a reflection that an optimal surgical strategy has not yet been reached. As the complication rate following chest wall reconstruction has been reported to range between 33 and 46 % [10, 13, 17], further innovation is necessary in order to improve outcomes. In this report, we present a technique for chest wall reconstruction using custom-designed titanium implants.

To date, smaller chest wall defects have been typically managed with local soft-tissue procedures and use of non-rigid graft materials. Soft-tissue techniques include skin grafting, local advancement flaps, pedicled flaps, or free flaps [8–11]. Additionally, a spectrum of prosthetic materials have been made available including polypropylene (Marlex^®^, Davol and Prolene^®^, Ethicon, Inc, Somerville, NJ, USA), polyester (Mersilene^®^, Ethicon, Inc.), polytetrafluorethylene (Gore-tex^®^ and Dualmesh^®^, W.L. Gore & Associates, Inc., Flagstaff, AZ, USA), and polyglactin-910 (Vicryl^®^, Ethicon, Inc.) [2, 8, 10, 12–17]. More recently, biologic meshes such as grafts from human dura [3] or porcine collagen matrices [18–21] have been developed with the theoretical advantage of allowing native tissue ingrowth. In our experience, some meshes such as polypropylene have demonstrated an affinity for developing dense adhesions, which may complicate additional surgical procedures. Currently, we prefer Gore-tex^®^ due to its strength, ease of manipulation, and minimal demonstrated adhesion development.

Reconstruction of larger chest wall defects often requires reconstruction with rigid components [13, 22]. However, caution must be exercised as rigid reconstructions have been reported to cause chronic pain and deformity, as well as have an increased infection risk [17, 23, 24]. Traditionally, rigid stability was provided with use of auto- or allogeneic rib grafts [6, 18, 20]. A variety of exogenous materials have also been developed to supplement rigid stability. One commonly used technique creates a molded prosthesis by sandwiching layers of synthetic graft material and methyl methacrylate cement [12, 14]. However, drawbacks of this technique include technical difficulty of fabrication, increased operative time, post-operative fracture, infection, and permanent rigid deformity [12–14, 17, 24]. Alternatively, titanium implants such as plates [16, 25, 26], mesh [27], or vertical expandable prosthetic titanium rib rods [12, 15, 18, 21, 25] have been described. A chest wall-specific prosthetic reconstruction system has also been developed (STRATOS™; MedXpert, GmbH, Heitersheim, Germany) [12, 14, 28].

The chest wall reconstruction technique described in the present study was developed for two female patients with large chest wall defects where it was felt that previous reconstruction techniques would not be capable of providing restoration of thoracic symmetry or allowing for staged breast reconstruction. In the design of this technique, we believe that the success of this reconstruction is contingent upon stable restoration of anterior to posterior thoracic continuity from the sternum to the remnant ribs or spine. While the aforementioned previously described reconstruction techniques have been utilized for successful reconstruction of large chest wall defects in other patients, none were specifically designed with the provision of restoring anterior to posterior thoracic continuity. Consequently, careful three-dimensional pre-operative planning is essential for the size and shape of the prosthesis. With both implants designed in this fashion, both patients in this series have demonstrated satisfactory clinical and functional outcomes after approximately 3 years and have undergone successful staged breast reconstruction procedures without evidence of implant collapse or subsidence. Additionally, it is also important that pre-operative planning includes skin or soft-tissue flap coverage needs due to the resultant increase in the size of the chest wall following placement of the implant. In the first patient with Poland’s syndrome, consultation with plastic surgery colleagues was obtained pre-operatively and latissimus flap closure was performed at the end of the procedure. However, flap closure was not necessary in the second case as the change in thoracic size was not as marked.

Chest wall deficiency has previously been associated with the development of scoliosis [2, 3, 19, 25, 29–31]. Factors that have been reported to increase the risk of the development of scoliosis include younger age, resection during periods of rapid growth, increased number of ribs resected, and posterior rib resections [30]. It has not been established if rigid chest wall reconstruction is capable of modifying the risk of scoliosis. In the patient with chest wall deficiency secondary to resection of osteosarcoma, the mildly progressive upper thoracic curve that had developed during the 5 years following her chest wall resection has not demonstrated further progression following the reconstruction.

The main weaknesses in this study are the limited number of patients and relatively short follow-up period. Complications are common following chest wall reconstruction and are reported to range from 33−46 %, with respiratory complications being the most frequent at 11−24 % [10, 13, 17]. While a majority of these occur in the early post-operative period, chest wall reconstructions are known to be susceptible to later failure secondary to the mobility of the thorax with constant respiratory motion. While no complications or failures were observed in a follow-up period of approximately 3 years in our two patients, this study is not able to make a determination of long-term safety. Nonetheless, we believe that this technique may be considered as a surgical option for reconstruction of large chest wall defects when restoration of chest wall symmetry and superior stability are desired.
